# Poly[diaqua­(μ_5_-1*H*-imidazole-4,5-di­carboxyl­ato)(μ_4_-1*H*-imidazole-4,5-di­carboxyl­ato)tris­ilver(I)ytterbium(III)]

**DOI:** 10.1107/S1600536812031303

**Published:** 2012-07-18

**Authors:** Si-Ming Zhu

**Affiliations:** a School of Light Industry and Food Science, South China University of Technology, Guangzhou 510641, People’s Republic of China

## Abstract

The asymmetric unit of the title compound, [Ag_3_Yb(C_5_HN_2_O_4_)_2_(H_2_O)_2_]_*n*_, contains three Ag^I^ ions, one Yb^III^ ion, two imidazole-4,5-dicarboxyl­ate ligands and two coordinating water mol­ecules. The Yb^III^ atom is eight-coordinated, in a bicapped trigonal prismatic coordination geometry, by six O atoms from three imidazole-4,5-dicarboxyl­ate ligands and two coordinating water mol­ecules. The two-coordinated Ag^I^ ions exhibit three types of coordination environments. One Ag^I^ atom is bonded to two N atoms from two different imidazole-4,5-dicarboxyl­ate ligands. The other two Ag^I^ atoms are each coordinated by one O atom and one N atom from two different imidazole-4,5-dicarboxyl­ate ligands. These metal coordination units are connected by bridging imidazole-4,5-dicarboxyl­ate ligands, generating a two-dimensional heterometallic layer. These layers are stacked along the *a* axis *via* O—H⋯O hydrogen-bonding inter­actions to generate a three-dimensional framework.

## Related literature

For the application of lanthanide–transition metal heterometallic complexes with bridging multifunctional organic ligands, see: Cheng *et al.* (2006 ▶); Kuang *et al.* (2007 ▶); Sun *et al.* (2006 ▶); Zhu *et al.* (2010 ▶).
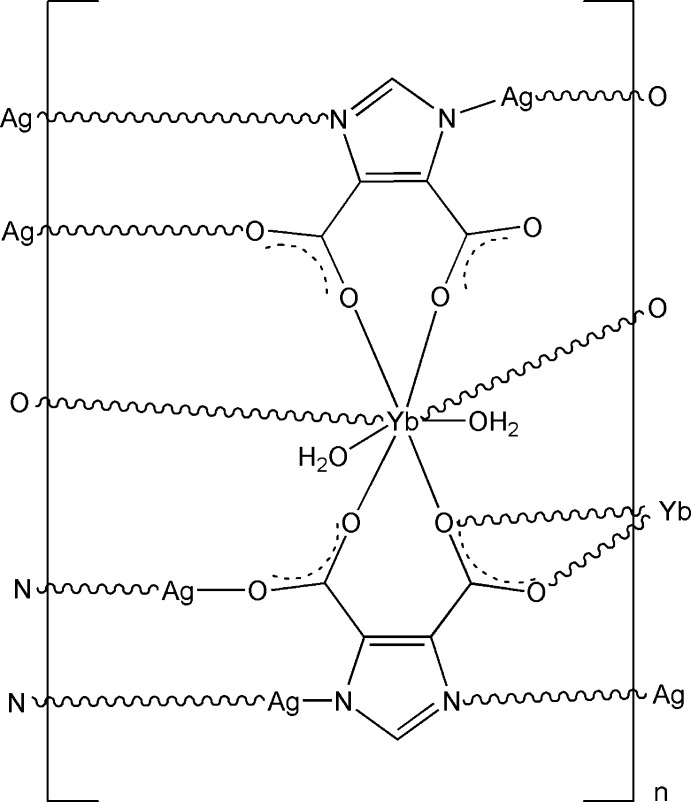


## Experimental

### 

#### Crystal data

[Ag_3_Yb(C_5_HN_2_O_4_)_2_(H_2_O)_2_]
*M* *_r_* = 838.84
Monoclinic, 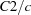

*a* = 12.6850 (7) Å
*b* = 8.6643 (5) Å
*c* = 28.4015 (16) Å
β = 97.686 (1)°
*V* = 3093.5 (3) Å^3^
*Z* = 8
Mo *K*α radiation
μ = 9.80 mm^−1^
*T* = 295 K
0.20 × 0.18 × 0.17 mm

#### Data collection

Bruker APEXII CCD diffractometer
Absorption correction: multi-scan (*SADABS*; Sheldrick, 1996 ▶) *T*_min_ = 0.162, *T*_max_ = 0.189
7613 measured reflections
2794 independent reflections
2629 reflections with *I* > 2σ(*I*)
*R*_int_ = 0.026

#### Refinement

*R*[*F* ^2^ > 2σ(*F* ^2^)] = 0.024
*wR*(*F* ^2^) = 0.055
*S* = 1.19
2794 reflections
265 parameters
4 restraints
H atoms treated by a mixture of independent and constrained refinement
Δρ_max_ = 0.58 e Å^−3^
Δρ_min_ = −1.29 e Å^−3^

### 

Data collection: *APEX2* (Bruker, 2004 ▶); cell refinement: *SAINT* (Bruker, 2004 ▶); data reduction: *SAINT*; program(s) used to solve structure: *SHELXS97* (Sheldrick, 2008 ▶); program(s) used to refine structure: *SHELXL97* (Sheldrick, 2008 ▶); molecular graphics: *SHELXTL* (Sheldrick, 2008 ▶); software used to prepare material for publication: *SHELXL97*.

## Supplementary Material

Crystal structure: contains datablock(s) I, global. DOI: 10.1107/S1600536812031303/rk2366sup1.cif

Structure factors: contains datablock(s) I. DOI: 10.1107/S1600536812031303/rk2366Isup2.hkl

Additional supplementary materials:  crystallographic information; 3D view; checkCIF report

## Figures and Tables

**Table 1:** Hydrogen-bond geometry (Å, °)

*D*—H⋯*A*	*D*—H	H⋯*A*	*D*⋯*A*	*D*—H⋯*A*
O1*W*—H1*W*⋯O8^i^	0.82 (2)	2.11 (5)	2.751 (5)	136 (6)
O1*W*—H2*W*⋯O2^ii^	0.81 (2)	2.03 (3)	2.823 (5)	165 (6)
O2*W*—H4*W*⋯O1^iii^	0.81 (2)	1.88 (4)	2.634 (5)	154 (8)

Symmetry codes: (i) 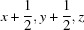; (ii) 
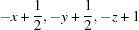
; (iii) 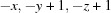.

**Fig. 1.**
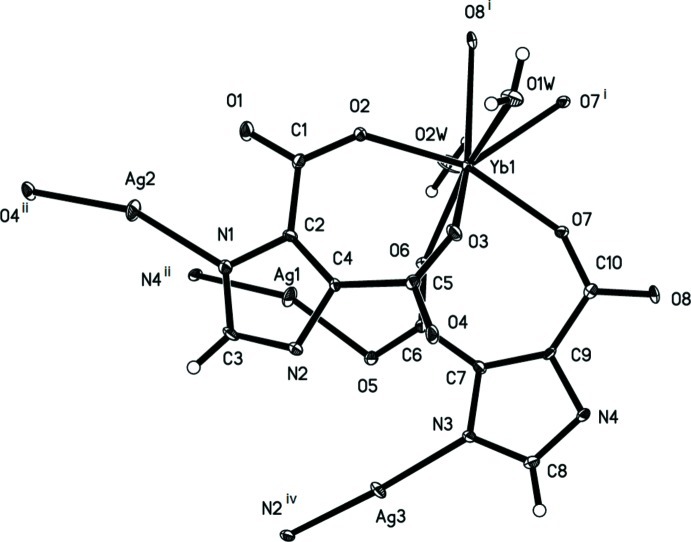
The molecular structure showing the atom numbering scheme. Displacement ellipsoids are drawn at the 30% probability level. H atoms are presented as a small spheres of arbitrary radius. Symmetry codes: (i) -x, -y, 1-z; (ii) x, 1+y, z; (iv) -x, y, 3/2-z.

**Fig. 2.**
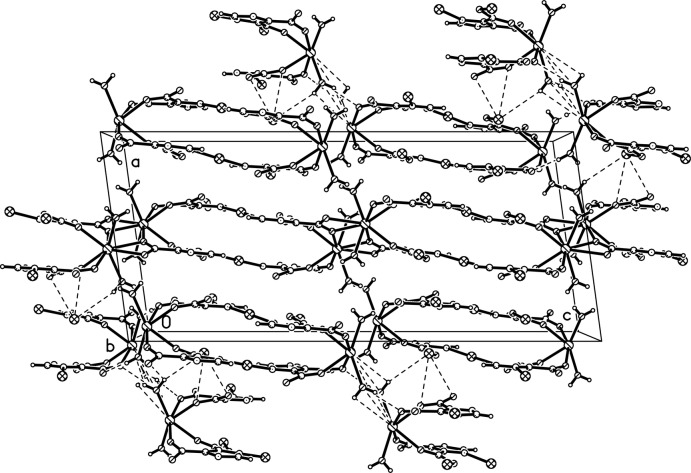
A view of the three-dimensional structure of the title compound. The hydrogen bonding interactions showed as broken lines.

## supplementary crystallographic information

### Comment

In the past few years, lanthanide-transition metal heterometallic complexs with bridging multifunctionnal organic ligands are of increasing interest, not only because of their impressive topological structures, but also due to their versatile applications in ion exchange, magnetism, bimetallic catalysis and luminescent probe (Cheng *et al.*, 2006; Kuang *et al.*, 2007; Sun *et al.*, 2006; Zhu *et al.*, 2010). As an extension of this research, the structure of the title compound, a new heterometallic coordination polymer, has been determined which is presented in this artcle.

The asymmetric unit of the title compound (Fig. 1), contains three Ag^I^ ions, one Yb^III^ ion, two imidazole-4,5-dicarboxylate ligands, and two coordinated water molecules. The Yb^III^ are eight-coordinated, in a bicapped trigonal prismatic coordination geometry, by six O atoms from three imidazole-4,5-dicarboxylate ligands and two coordinated water molecules. The two-coordinated Ag^I^ ions exhibit three types of coordination environment. One Ag^I^ ion is linear bonded to two N atoms from two different imidazole-4,5-dicarboxylate ligands with N2^iv^-Ag3-N3 angle 176.23 (17)°. The other two Ag^I^ ions are coordinated in a bow-like conformation each by one O atom and one N atom from two different imidazole-4,5-dicarboxylate ligands with N-Ag-O angle 157.45 (14)° and 159.80 (14)°, respectively. These metal coordination units are connected by bridging imidazole-4,5-dicarboxylate ligands, generating a two-dimensional heterometallic layer. The two-dimensional layers are stacked along *a* axis *via* O–H···O hydrogen-bonding interactions to generate the three-dimensional framework (Table 1 and Fig. 2). Symmetry code: (iv) -*x*, *y*, -*z*+3/2.

### Experimental

A mixture of AgNO_3_ (0.102 g, 0.6 mmol), Yb_2_O_3_ (0.118 g, 0.3 mmol), imidazole-4,5-dicarboxylic acid (0.188 g, 1.2 mmol), H_2_O (10 ml), and HClO_4_ (0.385 mmol) was sealed in a 20 ml teflon-lined reaction vessel at 443 K for 5 days then slowly cooled to room temperature. The product was collected by filtration, washed with water and air-dried. Colourless block crystals suitable for X-ray analysis were obtained.

### Refinement

H atoms bonded to C atoms were positioned geometrically and refined as riding, with C–H = 0.93Å and *U*_iso_(H) = 1.2*U*_eq_(C). H atoms of water molecules were found from difference Fourier maps and refined isotropically with a restraint of O–H = 0.82Å and *U*_iso_(H) = 1.5*U*_eq_(O).

### Crystal data

**Table:**

[Ag_3_Yb(C_5_HN_2_O_4_)_2_(H_2_O)_2_]	*F*(000) = 3080
*M**_r_* = 838.84	*D*_x_ = 3.602 Mg m^−^^3^
Monoclinic, *C*2/*c*	Mo *K*α radiation, λ = 0.71073 Å
Hall symbol: -C 2yc	Cell parameters from 4947 reflections
*a* = 12.6850 (7) Å	θ = 2.9–28.1°
*b* = 8.6643 (5) Å	µ = 9.80 mm^−^^1^
*c* = 28.4015 (16) Å	*T* = 295 K
β = 97.686 (1)°	Block, colourless
*V* = 3093.5 (3) Å^3^	0.20 × 0.18 × 0.17 mm
*Z* = 8	

### Data collection

**Table:**

Bruker APEXII CCD diffractometer	2794 independent reflections
Radiation source: fine-focus sealed tube	2629 reflections with *I* > 2σ(*I*)
Graphite monochromator	*R*_int_ = 0.026
φ and ω scan	θ_max_ = 25.2°, θ_min_ = 1.5°
Absorption correction: multi-scan (*SADABS*; Sheldrick, 1996)	*h* = −13→15
*T*_min_ = 0.162, *T*_max_ = 0.189	*k* = −10→8
7613 measured reflections	*l* = −34→34

### Refinement

**Table:**

Refinement on *F*^2^	Primary atom site location: structure-invariant direct methods
Least-squares matrix: full	Secondary atom site location: difference Fourier map
*R*[*F*^2^ > 2σ(*F*^2^)] = 0.024	Hydrogen site location: inferred from neighbouring sites
*wR*(*F*^2^) = 0.055	H atoms treated by a mixture of independent and constrained refinement
*S* = 1.19	*w* = 1/[σ^2^(*F*_o_^2^) + (0.0213*P*)^2^ + 8.4063*P*] where *P* = (*F*_o_^2^ + 2*F*_c_^2^)/3
2794 reflections	(Δ/σ)_max_ = 0.001
265 parameters	Δρ_max_ = 0.58 e Å^−^^3^
4 restraints	Δρ_min_ = −1.29 e Å^−^^3^

### Special details

**Table:**

Geometry. All s.u.'s (except the s.u. in the dihedral angle between two l.s. planes) are estimated using the full covariance matrix. The cell s.u.'s are taken into account individually in the estimation of s.u.'s in distances, angles and torsion angles; correlations between s.u.'s in cell parameters are only used when they are defined by crystal symmetry. An approximate (isotropic) treatment of cell s.u.'s is used for estimating s.u.'s involving l.s. planes.
Refinement. Refinement of *F*^2^ against ALL reflections. The weighted *R*-factor *wR* and goodness of fit *S* are based on *F*^2^, conventional *R*-factors *R* are based on *F*, with *F* set to zero for negative *F*^2^. The threshold expression of *F*^2^ > σ(*F*^2^) is used only for calculating *R*-factors(gt) *etc*. and is not relevant to the choice of reflections for refinement. *R*-factors based on *F*^2^ are statistically about twice as large as those based on *F*, and *R*-factors based on ALL data will be even larger.

### Fractional atomic coordinates and isotropic or equivalent isotropic displacement parameters (Å^2^)

**Table:**

	*x*	*y*	*z*	*U*_iso_*/*U*_eq_	
Yb1	0.062585 (17)	0.17662 (3)	0.534835 (7)	0.01157 (8)	
Ag1	−0.08531 (4)	0.52086 (5)	0.638040 (16)	0.02463 (12)	
Ag2	0.18074 (4)	0.78628 (5)	0.663984 (15)	0.02278 (12)	
Ag3	−0.12303 (3)	0.14446 (5)	0.739965 (13)	0.02025 (11)	
C1	0.1656 (4)	0.4861 (6)	0.59111 (17)	0.0128 (11)	
C2	0.1594 (4)	0.4326 (6)	0.64091 (16)	0.0114 (10)	
C3	0.1486 (4)	0.4663 (6)	0.71522 (17)	0.0163 (11)	
H3	0.1434	0.5143	0.7441	0.020*	
C4	0.1601 (4)	0.2898 (6)	0.66262 (17)	0.0119 (10)	
C5	0.1733 (4)	0.1266 (6)	0.64641 (17)	0.0120 (11)	
C6	−0.0760 (4)	0.1879 (6)	0.62733 (17)	0.0121 (11)	
C7	−0.0744 (4)	0.0214 (6)	0.64145 (16)	0.0124 (11)	
C8	−0.0872 (4)	−0.1680 (6)	0.68906 (18)	0.0173 (12)	
H8	−0.0933	−0.2230	0.7167	0.021*	
C9	−0.0685 (4)	−0.1163 (6)	0.61662 (17)	0.0114 (10)	
C10	−0.0598 (4)	−0.1506 (6)	0.56693 (17)	0.0121 (11)	
O1	0.1765 (3)	0.6248 (4)	0.58442 (12)	0.0255 (10)	
O2	0.1559 (3)	0.3874 (4)	0.55739 (12)	0.0172 (8)	
O3	0.1588 (3)	0.0949 (4)	0.60304 (12)	0.0169 (8)	
O4	0.1991 (3)	0.0300 (4)	0.67886 (12)	0.0196 (8)	
O5	−0.1075 (3)	0.2825 (4)	0.65620 (13)	0.0200 (8)	
O6	−0.0463 (3)	0.2298 (4)	0.58910 (12)	0.0171 (8)	
O7	−0.0256 (3)	−0.0485 (4)	0.54003 (11)	0.0164 (8)	
O8	−0.0893 (3)	−0.2776 (4)	0.54873 (12)	0.0193 (8)	
N1	0.1517 (3)	0.5441 (5)	0.67471 (14)	0.0148 (9)	
N2	0.1537 (4)	0.3137 (5)	0.71038 (14)	0.0149 (9)	
N3	−0.0869 (3)	−0.0151 (5)	0.68748 (14)	0.0140 (9)	
N4	−0.0780 (3)	−0.2366 (5)	0.64767 (14)	0.0142 (9)	
O1W	0.2245 (3)	0.0680 (5)	0.51848 (13)	0.0221 (9)	
H1W	0.275 (4)	0.079 (8)	0.5390 (17)	0.033*	
H2W	0.249 (5)	0.075 (8)	0.4936 (13)	0.033*	
O2W	−0.0917 (4)	0.2942 (6)	0.50178 (14)	0.0347 (11)	
H4W	−0.130 (5)	0.298 (9)	0.4768 (15)	0.052*	
H3W	−0.138 (5)	0.331 (8)	0.515 (3)	0.052*	

### Atomic displacement parameters (Å^2^)

**Table:**

	*U*^11^	*U*^22^	*U*^33^	*U*^12^	*U*^13^	*U*^23^
Yb1	0.01795 (13)	0.01015 (13)	0.00699 (12)	−0.00344 (9)	0.00307 (8)	−0.00139 (8)
Ag1	0.0334 (3)	0.0086 (2)	0.0326 (3)	−0.00032 (18)	0.00726 (19)	0.00049 (18)
Ag2	0.0308 (3)	0.0085 (2)	0.0290 (2)	−0.00071 (17)	0.00390 (19)	−0.00148 (17)
Ag3	0.0304 (3)	0.0191 (2)	0.0125 (2)	0.00166 (18)	0.00737 (17)	−0.00609 (16)
C1	0.015 (3)	0.010 (3)	0.012 (2)	−0.003 (2)	−0.001 (2)	0.001 (2)
C2	0.015 (3)	0.011 (3)	0.008 (2)	0.001 (2)	0.0039 (19)	0.000 (2)
C3	0.027 (3)	0.015 (3)	0.008 (2)	0.002 (2)	0.007 (2)	−0.003 (2)
C4	0.020 (3)	0.008 (3)	0.009 (2)	−0.002 (2)	0.004 (2)	−0.0018 (19)
C5	0.010 (2)	0.012 (3)	0.014 (3)	−0.002 (2)	0.0024 (19)	−0.001 (2)
C6	0.016 (3)	0.010 (3)	0.010 (2)	−0.002 (2)	0.001 (2)	−0.001 (2)
C7	0.018 (3)	0.011 (3)	0.008 (2)	0.003 (2)	0.0023 (19)	−0.0003 (19)
C8	0.026 (3)	0.012 (3)	0.015 (3)	0.000 (2)	0.009 (2)	0.003 (2)
C9	0.012 (3)	0.008 (3)	0.015 (2)	−0.003 (2)	0.004 (2)	0.002 (2)
C10	0.011 (3)	0.011 (3)	0.013 (2)	−0.001 (2)	−0.001 (2)	0.001 (2)
O1	0.052 (3)	0.010 (2)	0.0124 (19)	−0.0062 (18)	−0.0007 (18)	0.0020 (15)
O2	0.030 (2)	0.014 (2)	0.0091 (17)	−0.0080 (16)	0.0047 (15)	−0.0025 (15)
O3	0.026 (2)	0.015 (2)	0.0093 (17)	0.0017 (16)	0.0018 (14)	−0.0024 (15)
O4	0.039 (2)	0.0067 (19)	0.0121 (18)	0.0035 (17)	0.0004 (16)	−0.0005 (15)
O5	0.035 (2)	0.009 (2)	0.0194 (19)	0.0001 (16)	0.0143 (17)	−0.0007 (16)
O6	0.026 (2)	0.014 (2)	0.0124 (18)	−0.0004 (16)	0.0070 (15)	0.0023 (15)
O7	0.026 (2)	0.015 (2)	0.0091 (17)	−0.0094 (16)	0.0056 (15)	−0.0020 (15)
O8	0.029 (2)	0.013 (2)	0.0175 (19)	−0.0060 (16)	0.0063 (16)	−0.0095 (16)
N1	0.022 (2)	0.011 (2)	0.011 (2)	−0.0014 (19)	0.0023 (17)	−0.0002 (18)
N2	0.024 (2)	0.013 (2)	0.008 (2)	−0.0016 (18)	0.0048 (17)	−0.0012 (17)
N3	0.023 (2)	0.010 (2)	0.010 (2)	0.0014 (18)	0.0071 (18)	0.0000 (17)
N4	0.022 (2)	0.007 (2)	0.015 (2)	0.0018 (18)	0.0071 (18)	0.0032 (17)
O1W	0.019 (2)	0.032 (2)	0.015 (2)	−0.0022 (18)	0.0042 (15)	−0.0015 (18)
O2W	0.039 (3)	0.052 (3)	0.014 (2)	0.024 (2)	0.0048 (18)	0.008 (2)

### Geometric parameters (Å, º)

**Table:**

Yb1—O2	2.224 (4)	C4—N2	1.385 (6)
Yb1—O6	2.251 (3)	C4—C5	1.504 (7)
Yb1—O3	2.261 (3)	C5—O3	1.251 (6)
Yb1—O7	2.263 (3)	C5—O4	1.255 (6)
Yb1—O2W	2.293 (4)	C6—O6	1.249 (6)
Yb1—O1W	2.361 (4)	C6—O5	1.262 (6)
Yb1—O7^i^	2.389 (3)	C6—C7	1.496 (7)
Yb1—O8^i^	2.594 (4)	C7—N3	1.375 (6)
Yb1—C10^i^	2.895 (5)	C7—C9	1.393 (7)
Yb1—Yb1^i^	3.8682 (5)	C8—N3	1.326 (7)
Ag1—N4^ii^	2.119 (4)	C8—N4	1.336 (7)
Ag1—O5	2.157 (4)	C8—H8	0.9300
Ag2—N1	2.159 (4)	C9—N4	1.381 (6)
Ag2—O4^ii^	2.160 (4)	C9—C10	1.461 (7)
Ag2—Ag3^iii^	3.3055 (6)	C10—O8	1.251 (6)
Ag3—N2^iv^	2.107 (4)	C10—O7	1.283 (6)
Ag3—N3	2.127 (4)	C10—Yb1^i^	2.895 (5)
Ag3—Ag3^iv^	3.0969 (9)	O4—Ag2^vi^	2.160 (3)
Ag3—Ag2^v^	3.3055 (6)	O7—Yb1^i^	2.389 (3)
C1—O1	1.228 (6)	O8—Yb1^i^	2.594 (4)
C1—O2	1.277 (6)	N2—Ag3^iv^	2.107 (4)
C1—C2	1.500 (7)	N4—Ag1^vi^	2.119 (4)
C2—N1	1.375 (6)	O1W—H1W	0.82 (2)
C2—C4	1.382 (7)	O1W—H2W	0.81 (2)
C3—N2	1.331 (7)	O2W—H4W	0.81 (2)
C3—N1	1.339 (7)	O2W—H3W	0.81 (2)
C3—H3	0.9300		
			
O2—Yb1—O6	89.21 (13)	N1—C2—C4	108.3 (4)
O2—Yb1—O3	78.74 (13)	N1—C2—C1	117.3 (4)
O6—Yb1—O3	77.75 (13)	C4—C2—C1	134.4 (5)
O2—Yb1—O7	159.69 (12)	N2—C3—N1	113.8 (4)
O6—Yb1—O7	77.17 (13)	N2—C3—H3	123.1
O3—Yb1—O7	83.64 (13)	N1—C3—H3	123.1
O2—Yb1—O2W	98.34 (17)	C2—C4—N2	107.8 (4)
O6—Yb1—O2W	67.73 (13)	C2—C4—C5	134.4 (4)
O3—Yb1—O2W	145.43 (14)	N2—C4—C5	117.6 (4)
O7—Yb1—O2W	90.43 (17)	O3—C5—O4	124.5 (5)
O2—Yb1—O1W	86.59 (14)	O3—C5—C4	120.0 (4)
O6—Yb1—O1W	147.79 (13)	O4—C5—C4	115.5 (4)
O3—Yb1—O1W	70.10 (13)	O6—C6—O5	122.3 (5)
O7—Yb1—O1W	96.89 (14)	O6—C6—C7	121.2 (4)
O2W—Yb1—O1W	144.46 (13)	O5—C6—C7	116.5 (4)
O2—Yb1—O7^i^	132.22 (12)	N3—C7—C9	107.8 (4)
O6—Yb1—O7^i^	129.72 (12)	N3—C7—C6	118.5 (4)
O3—Yb1—O7^i^	129.53 (13)	C9—C7—C6	133.6 (4)
O7—Yb1—O7^i^	67.50 (13)	N3—C8—N4	114.5 (5)
O2W—Yb1—O7^i^	77.69 (15)	N3—C8—H8	122.8
O1W—Yb1—O7^i^	73.28 (13)	N4—C8—H8	122.8
O2—Yb1—O8^i^	81.79 (11)	N4—C9—C7	107.9 (4)
O6—Yb1—O8^i^	136.44 (12)	N4—C9—C10	119.2 (4)
O3—Yb1—O8^i^	140.18 (13)	C7—C9—C10	132.8 (4)
O7—Yb1—O8^i^	118.45 (11)	O8—C10—O7	117.8 (4)
O2W—Yb1—O8^i^	71.58 (14)	O8—C10—C9	121.4 (5)
O1W—Yb1—O8^i^	74.40 (13)	O7—C10—C9	120.7 (4)
O7^i^—Yb1—O8^i^	51.43 (11)	O8—C10—Yb1^i^	63.6 (3)
O2—Yb1—C10^i^	106.65 (13)	O7—C10—Yb1^i^	54.5 (2)
O6—Yb1—C10^i^	140.62 (13)	C9—C10—Yb1^i^	171.2 (4)
O3—Yb1—C10^i^	139.85 (13)	C1—O2—Yb1	139.5 (3)
O7—Yb1—C10^i^	93.32 (13)	C5—O3—Yb1	140.0 (3)
O2W—Yb1—C10^i^	74.33 (14)	C5—O4—Ag2^vi^	119.8 (3)
O1W—Yb1—C10^i^	70.57 (13)	C6—O5—Ag1	113.8 (3)
O7^i^—Yb1—C10^i^	25.90 (12)	C6—O6—Yb1	145.0 (3)
O8^i^—Yb1—C10^i^	25.60 (12)	C10—O7—Yb1	147.5 (3)
O2—Yb1—Yb1^i^	164.48 (9)	C10—O7—Yb1^i^	99.6 (3)
O6—Yb1—Yb1^i^	105.35 (9)	Yb1—O7—Yb1^i^	112.50 (13)
O3—Yb1—Yb1^i^	109.17 (9)	C10—O8—Yb1^i^	90.8 (3)
O7—Yb1—Yb1^i^	34.79 (8)	C3—N1—C2	105.0 (4)
O2W—Yb1—Yb1^i^	82.69 (13)	C3—N1—Ag2	129.5 (4)
O1W—Yb1—Yb1^i^	83.82 (10)	C2—N1—Ag2	123.7 (3)
O7^i^—Yb1—Yb1^i^	32.71 (8)	C3—N2—C4	105.0 (4)
O8^i^—Yb1—Yb1^i^	83.89 (8)	C3—N2—Ag3^iv^	127.3 (3)
C10^i^—Yb1—Yb1^i^	58.56 (10)	C4—N2—Ag3^iv^	126.3 (3)
N4^ii^—Ag1—O5	157.46 (14)	C8—N3—C7	105.3 (4)
N1—Ag2—O4^ii^	159.80 (14)	C8—N3—Ag3	128.6 (3)
N1—Ag2—Ag3^iii^	70.99 (11)	C7—N3—Ag3	125.5 (3)
O4^ii^—Ag2—Ag3^iii^	100.58 (10)	C8—N4—C9	104.6 (4)
N2^iv^—Ag3—N3	176.23 (17)	C8—N4—Ag1^vi^	123.2 (3)
N2^iv^—Ag3—Ag3^iv^	98.55 (12)	C9—N4—Ag1^vi^	132.2 (3)
N3—Ag3—Ag3^iv^	79.79 (12)	Yb1—O1W—H1W	116 (5)
N2^iv^—Ag3—Ag2^v^	89.30 (12)	Yb1—O1W—H2W	126 (5)
N3—Ag3—Ag2^v^	89.89 (11)	H1W—O1W—H2W	105 (6)
Ag3^iv^—Ag3—Ag2^v^	140.588 (19)	Yb1—O2W—H4W	140 (6)
O1—C1—O2	122.7 (5)	Yb1—O2W—H3W	128 (6)
O1—C1—C2	118.0 (5)	H4W—O2W—H3W	90 (7)
O2—C1—C2	119.2 (4)		

Symmetry codes: (i) −*x*, −*y*, −*z*+1; (ii) *x*, *y*+1, *z*; (iii) *x*+1/2, *y*+1/2, *z*; (iv) −*x*, *y*, −*z*+3/2; (v) *x*−1/2, *y*−1/2, *z*; (vi) *x*, *y*−1, *z*.

### Hydrogen-bond geometry (Å, º)

**Table:**

*D*—H···*A*	*D*—H	H···*A*	*D*···*A*	*D*—H···*A*
O1*W*—H1*W*···O8^iii^	0.82 (2)	2.11 (5)	2.751 (5)	136 (6)
O1*W*—H2*W*···O2^vii^	0.81 (2)	2.03 (3)	2.823 (5)	165 (6)
O2*W*—H4*W*···O1^viii^	0.81 (2)	1.88 (4)	2.634 (5)	154 (8)

Symmetry codes: (iii) *x*+1/2, *y*+1/2, *z*; (vii) −*x*+1/2, −*y*+1/2, −*z*+1; (viii) −*x*, −*y*+1, −*z*+1.
